# The Moderating Effect of Store Format on the Relationships Between ICT, Innovation and Sustainability in Retailing

**DOI:** 10.3389/fpsyg.2021.678991

**Published:** 2021-05-06

**Authors:** Antonio Marín-García, Irene Gil-Saura, María-Eugenia Ruiz-Molina, Gloria Berenguer-Contrí

**Affiliations:** Facultad de Economía, Universidad de Valencia, Valencia, Spain

**Keywords:** ICT, innovation, sustainability, retailing, Artificial Intelligence

## Abstract

Innovation and sustainability are postulated as key variables for the future of large commercial distribution. In addition, the development of Information and Communication Technologies (ICT) solutions, and especially those related to Artificial Intelligence (i.e., Just Walk Out, Intelligent Retail Lab) and digitization, are particularly relevant factors in the current pandemic scenario in which retail companies operate. These tools are essential to face the derived changes in commercial relations, especially between companies and consumers. For all these reasons, this work aims to examine the effect of ICT, as a driving factor for innovation and its direct and indirect impact on sustainability in retailing. Furthermore, this study takes into account the eventual differences in these relationships according to the types of store formats. To achieve the aforementioned objective, a theoretical model is proposed that is tested through an empirical study carried out on a sample of 510 consumers of three store formats of grocery retail formats (i.e., hypermarkets, supermarkets and discount stores) in Spain. For the analysis of the data obtained, the partial least squares (PLS) regression technique and the Multigroup Analysis were used. The results obtained confirm the direct and indirect effect of ICT on innovation and sustainability in grocery retailing. In addition, the results indicate that consumers unequally perceive the technological progress of companies. These differences are greater between discount stores versus hypermarkets and supermarkets. The larger the size of the store format, the higher the incidence of ICT in relation to innovation. Therefore, it is vital to take ICT into consideration, where Artificial Intelligence is imperative for the growth and development of sustainable competitive advantages in retail companies.

## Introduction

Advances in the market digitization process as a result of environmental changes have been noticeable in recent years. Information and Communication Technologies (ICT) have been one of the main factors that has modified the way companies relate to consumers, especially with the development of Artificial Intelligence (AI) (Rodgers et al., 2021). In addition, the global crisis caused by COVID-19 has accelerated the digitization process of companies and today is one of the main priorities on the agendas of most managers. Undoubtedly, the pandemic has put thousands of traditional businesses to the test; after closing their doors to the public, they were forced to resort to new ways of attracting consumers and marketing their products, with the aim of remaining operational and simply surviving. But digitalization is a process in which different retailers may be at a different level of evolution, and where the incorporation of artificial intelligence is an additional step that can bring new opportunities in the relationship with customers.

As a consequence of the above, many companies find themselves in the position of incorporating new, more sophisticated and innovative tools in their daily activities, directly linked to ICT, among which is Artificial Intelligence (Dubey et al., 2019; Mahmoud et al., 2020; Rodgers et al., 2021). Retailing, one of the sectors most affected by the economic crisis brought about by COVID-19 with a nearly 18% decrease in sales in Spain (Instituto Nacional de Estadística [INE], 2021), identifies the incorporation of ICT in its businesses as an environmental opportunity. In this sense, these tools are positioned as a strategic axis on which to lean in order to survive and be more competitive.

Taking this new approach as a reference, some studies propose that the future of retail has to be approached from a dual perspective that is innovative and sustainable (Marcon et al., 2017; Marín-García et al., 2020, 2021). Innovation and sustainability are considered the main pillars on which the restructuring of retailing must be based. In addition, these factors are identified as key elements for the development of competitive advantages and for the survival of companies (Gonzalez-Lafaysse and Lapassouse-Madrid, 2016; Morioka et al., 2016; Lüdeke-Freund et al., 2017). In this sense, some studies suggest that those retail formats that implement innovative and sustainable actions in their daily activities will ensure that their customers are more satisfied, and consequently retain these customer (Gonzalez-Lafaysse and Lapassouse-Madrid, 2016; Marcon et al., 2017; Marín-García et al., 2020, 2021). However, in light of existing studies, the benefits obtained by retail companies thanks to the implementation of sustainable and innovative practices may be affected by the nature of the store format (Catuogno and Gutierres, 2015; Filipe et al., 2017).

In this sense, the study of innovation and sustainability in retailing, driven by ICT, emerges as an area of preferential interest. The analysis of consumer perceptions about the degree of technological advancement of companies, and the implementation of actions related to sustainability and innovation in retail businesses acquire a capital of interest. This interest is higher if we consider the current environment in which companies in the commercial sector operate. However, in the context of the retail sector and, from the consumer’s point of view, there is still little research going in that direction. Therefore, this work proposes the analysis of ICT as a dynamic element of innovation and sustainability in retail. Furthermore, this research aims to examine both the direct and indirect impact of ICT on sustainability, through the mediating effect of innovation. Finally, this study also addresses the moderating effect of store format to observe the possible differences in the relationships raised, all from the consumer’s point of view. With all this, this work will be approached considering that line of research that shows that the progress of the retailing is based on two fundamental pillars, innovation and sustainability (Gonzalez-Lafaysse and Lapassouse-Madrid, 2016; Morioka et al., 2016; Marcon et al., 2017; Marín-García et al., 2020, 2021). These works defend that the current business model in retail commercial distribution must be built from a double perspective, if it is to ensure success in the market: commerce must be innovative and sustainable. For this, the study of innovation will be examined from the approach proposed by Musso (2010) and Lin (2015), while the analysis of sustainability will be carried out considering the Triple Bottom Line theory proposed by Elkington (2004).

To achieve the main objectives of this research, it will proceed in different stages. First, after the introduction, the main constructs of this study will be addressed theoretically. Specifically, this work will focus on ICT, with particular attention to AI, innovation and sustainability in retail, taking the consumer’s perspective as an approach for analysis. Subsequently, the hypotheses that will shape the relationships of the theoretical model will be formulated. Thirdly, the methodology for the empirical study that guides this work will be explained and fourthly we will present the results obtained. Finally, after presenting the main conclusions of the study, we will explain the implications for management that could derive from these results, as well as the main limitations and future lines of research.

## Theoretical Framework

### ICT in Retail

The review of the literature has allowed us to observe that the study of ICT has been one of the topics to which the most attention has been paid among researchers from different study areas (Gil-Saura et al., 2014; Ruiz-Molina et al., 2017). Traditionally, ICT have been associated with the set of *“applications that are basically used inside the organization are office automation, transaction processing system, enterprise resource planning systems, data warehousing systems, groupware applications, intranets, and executive information systems”* – (Ryssel et al., 2004: 198). At present, ICT have been recognized as a relevant aspect in the innovation process of companies, including retailers, in addition to being considered as one of the main contributors to business success (Ham et al., 2005). These types of tools are essential for the development of competitive advantages as they offer solutions in management and in the development of products and services, generating added value for customers (Ruiz-Molina et al., 2020).

There are numerous studies that have examined the advantages that ICT bring to the management of companies, as well as being a fundamental pillar for the development of innovative processes in organizations (MahbubulHye et al., 2020). In this sense, numerous conceptual and empirical works attempt to explain the links that exist between ICT and innovation (Wu et al., 2006; Musso, 2010; Reinartz et al., 2011; Lin and Wu, 2011). Here, ICT are presented as a key element in the innovative actions developed by retailers, as the clear need to pay greater attention to relationships with the different members of the distribution channels, especially customers, is observed. On the other hand, ICT are postulated as a facilitating element for the effective development of knowledge and innovation, which are decisive factors in the economic growth of retail companies.

The development of many of these technological innovations includes electronic commerce, point of sale (POS) terminal, bar code systems, optical readers, and electronic data interchange (EDI) systems, that is, ICT directly linked to the end consumer, which involve the implementation of information technology throughout the product development process, up until its sale to the end consumer (MahbubulHye et al., 2020; Mahmoud et al., 2020). All these technologies are grouped together under what is currently called Artificial Intelligence, among which the development of virtual assistants, intelligent robotics and the disappearance of tills are of particular note (Mahmoud et al., 2020; Rodgers et al., 2021). In this way, the use of ICT allows companies to obtain important information about consumers, such as their needs, expectations and purchasing behaviors, which contribute to the creation of innovation in retailing, and which allows for a more tailored service to customers (Ruiz-Molina et al., 2020). Likewise, it seems clear that companies with a powerful innovative character are more inclined to implement and use ICT in their businesses. Furthermore, the implementation of ICT in retail formats has a direct impact both from the point of view of the company and on consumer behaviors. For all these reasons, ICT are considered a cornerstone in the development of competitive advantages for retail companies due to their ability to reduce costs, increase market share and ramp up customer satisfaction (Gil-Saura et al., 2010) or improve the perception that consumers regarding to the image of the store, in addition to raise their purchase intentions (Cervantes and Franco, 2020).

The use of Artificial Intelligence in retail is progressively becoming a primary issue, supported by the take-off of online shopping, the development of new consumer habits and the search for a business model with characteristics similar to that of discount stores (Liu et al., 2018; Mahmoud et al., 2020). In this way, the retail sector is rapidly turning to the use of machine intelligence to efficiently simulate human intelligence and increase competitiveness by reducing cost and improving the customer experience (Dubey et al., 2019; Mahmoud et al., 2020). In this sense, some authors attempt to explain the shopping experience of consumers in those businesses that implement ICT directly linked to AI (Liu et al., 2018). However, the contributions in this line of research are still scarce, and studies that try to explain the main advantages and disadvantages of this type of tool, both from the point of view of companies and consumers are still at an exploratory level (Semenov et al., 2017; Liu et al., 2018; Mahmoud et al., 2020; Rodgers et al., 2021).

### Innovation

Innovation has been defined as a key aspect for the economic and competitive development of companies (Aramburu et al., 2015; Marín-García and Gil-Saura, 2017; Olsson et al., 2019; Marín-García et al., 2020). In this sense, innovation is presented as a capital factor for the creation of wealth and the growth of economies, which allows companies to access new segments, be more competitive, and guarantee their expansion (Dawson and Frasquet, 2006).

Additionally, innovation is a key element for the long-term survival of companies, even when the market environment in which they operate is characterized by high complexity and turbulence (Hernández-Espallardo et al., 2011). Lin and Wu (2011) approach innovation in the field of companies from a strategic perspective, highlighting that innovation is fundamental in the corporate strategy of companies. This vision is shared by Musso (2010), who, from the perspective of retailers, adds that innovation in distribution channels has a dual purpose. On the one hand, it should be seen as a strategic activity, not only for distribution companies but also for industrial organizations, establishing the acquisition of competitive advantages as one of its main objectives. On the other hand, innovation in distribution channels must act as a catalyst, that is, as the key factor to promote changes in the economic function of distribution systems.

Innovation in the context of retail is a construct that began to acquire capital interest in the 1990s, thanks, in part, to the evolution and development of new technologies. Until that moment, innovation in commercial distribution had been mainly associated with the changes produced as a consequence of the evolution of store formats (Dawson and Frasquet, 2006; Moliner-Velázquez et al., 2019). However, at present, the study of innovation is starting to be approached from other perspectives, giving greater prominence to the evolution of the product, the brand, the pricing models and the relationships between the members of the channel. All this, considering the consumer as the cornerstone of this evolution. In addition, the innovation of retail businesses has been observed conceptually in response to the globalized environment of the markets. Thus, Reinartz et al. (2011) consider the effect of globalization to explain the changes produced in the supply chain, the product assortment, the store format, and the brand itself, both of the product and the retail formats.

Identifying and defining the different types of innovation has not been an easy task in the field of marketing. One of the most referenced groupings in the literature is the one that distinguishes between technological innovations and non-technological innovations (Lin, 2015; Stagnaro, 2017; De Oliveira et al., 2020; Kim et al., 2020). Technological innovations include: (a) product innovations; and, (b) process innovations; while non-technological innovations group together: (a) organizational innovations; (b) marketing innovations; and, (c) relational innovations. Product innovations are understood as improvements or variations in existing products, or the introduction of new products that have not been marketed to date (Blanco-Callejo and de Pablos-Heredero, 2019). Process innovations consist of the implementation or adoption of a production method that may include changes in equipment, human resources or working methods (Liu and De Giovanni, 2019). Considering the non-technological innovations, the organizational ones imply the formulation of new strategies and organizational forms that directly or indirectly affect the basic activities inherent to a company’s business (Olsson et al., 2019). On the other hand, marketing innovations are defined as alterations that occur in the design or packaging, positioning, promotion or pricing criteria, in the marketing of a product or service (Quaye and Mensah, 2019). Lastly, relational innovation is linked to improving trust, loyalty and the quality of relationships between the parties involved (Marín-García et al., 2020).

The approach we adopt in this work considers innovation from the perspective presented by Lin (2015), who postulates a triple-pronged approach to the concept of innovation in marketing: product innovation, marketing innovation, and relational innovation. In addition, following this author’s proposal, the concept of innovation will be approached from the consumer’s perspective.

### Sustainability

Interest in studying the concept of sustainability emerged in the late 1980s with the presentation of the Brundtland Report (Chow and Chen, 2012; Lavorata, 2014; Ruiz-Real et al., 2019). This document manifested the need to consider the impact that economic progress was having on the environment, with a change in the business models of large companies deemed vital (Quaye and Mensah, 2019).

From that moment, a movement began, led by those studies that questioned traditional business models and were supported by a large part of society that was gradually becoming more aware of the need to care for the environment. As a consequence, companies and public institutions see that they must make strategic decisions regarding the evolution of their business models (Morioka et al., 2016; Kumar et al., 2017; De Vass et al., 2020), and that sustainable development is an essential element (Elkington, 2004; Kamara et al., 2006; Ruiz-Real et al., 2019; Marín-García et al., 2021). Lüdeke-Freund (2010): (1) describes the sustainable business model as “*business model eco-innovation should create competitive advantage through superior customer value (strategic requirement) and contribute to a sustainable development of the company and society*.”

However, in the retail context, in recent years the concept of sustainability has acquired a special role, especially since it is considered as an element closely linked to innovation. Some studies show how innovation is key to the transition from traditional business models that have prevailed in recent decades, to sustainable business models (Morioka et al., 2016; Lüdeke-Freund et al., 2017; Marcon et al., 2017). These new business models are mainly characterized by the reduction in the impact that economic activity inflicts on the environment and society (Morioka et al., 2016; Lüdeke-Freund et al., 2017).

With this new understanding of business models, the Triple Bottom Line, a term coined by Elkington (2004), was born. This author states that business success will depend on the ability of companies to add environmental, social and economic values to their daily activities, including three key concepts around them: planet, people and profit. Therefore, taking into account these three pillars of sustainability, environmental value has been explained as the actions developed by companies to create products and services without causing harm to the environment (Bakos et al., 2020). The social dimension is associated with the ability of companies to manage their businesses, improve quality of life and reinforce the relationships that organizations have with the various stakeholders that make up their environment (Malak-Rawlikowska et al., 2019). Finally, the economic dimension is essential, as it is considered a key requirement for the survival of companies (De Vass et al., 2020).

Following the development perspective of this work, from the consumer’s point of view, and paying attention to studies that have focused on the concept of sustainable development in retail businesses, this research considers the three dimensions of sustainability identified by Elkington (2004): environmental, social, and economic.

### Formulation of Hypotheses and Presentation of the Conceptual Model

#### Relationship Between ICT and Innovation

One of the main reasons for economic growth in retail is a consequence of the development of resources and capacities, such as knowledge and innovation (Beata, 2016). These factors facilitate the implementation of innovative solutions based on information and communication technologies (Reinartz et al., 2011; Arvanitis et al., 2013; De Oliveira et al., 2020).

In retailing, it has been observed how the development and implementation of ICT favor the reduction of the risk of decision-making related to innovation. When a company introduces new products to the market, in addition to the speed with which it is carried out, it is also essential to increase the precision and reduce the errors that accompany this process, and that can entail great costs for organizations. As a consequence of the growth of global brands and the increasingly massive and dynamic transfer of information, it is essential to develop and implement ICT tools to support the introduction of innovative products and services (Arvanitis et al., 2013; Beata, 2016; Marín-García and Gil-Saura, 2017).

Continuing along the same lines, the retail sector, works such as that of Vilaseca-Requena et al. (2007) reflect that the use of ICT favors the predisposition of companies when it comes to integrating particular agents within the business environment. All this allows the development of innovation processes, and helps to improve consumer acceptance of new products and/or services. In addition, the use of ICT favors the development of process innovation, which in turn makes it possible to improve the degree of product adaptation. ICT exert a positive effect in order to increase and improve the perception that consumers have of the innovations made by businesses.

The first of our hypotheses is supported by these arguments and therefore we formulate the following:

H1: *The use of ICT in retail has a positive and significant effect on customer perceptions of innovative practices in the store.*

#### Relationship Between ICT and Sustainability

In recent years, the study of the use of ICT in organizations and its impact on the environment and society is gaining great relevance (Wang et al., 2015; De Vass et al., 2020). Today, most companies in the retail sector operate in highly competitive environments, where intensive use of resources is common. In this sense, some studies try to examine the impact of ICT on aspects related to sustainability and with this analyse whether or not this use of ICT is helping retail companies to be sustainable. Despite the incipient interest, a large part of the studies that address this relationship do so from an exploratory and qualitative perspective.

In the context of retail, Wang et al. (2015) try to empirically analyse how ICT can contribute to the reduction of CO_2_ emissions in road freight transport. The result of their study confirms the positive impact that ICT have in reducing CO_2_ emissions. However, the authors point out that this benefit could be greater if the companies entered into joint collaboration commitments to address these emissions.

De Vass et al. (2020) also examine the relationship between ICT and sustainability. In this sense, the authors point out the importance of new technologies in reducing production costs, saving time in the execution of activities, and the benefits for companies from a triple perspective: financial, social and environmental.

On the other hand, Gonzalez-Lafaysse and Lapassouse-Madrid (2016), from the consumer perspective, highlight the benefits of using ICT as a means of support for companies in their work to communicate actions related to sustainability. In this sense, the authors suggest that the use of digital social media can reach consumers and communicate the sustainable practices implemented by the company. Gonzalez-Lafaysse and Lapassouse-Madrid (2016) point out that for this communication to be effective, it is necessary that the messages transmitted are not perceived by consumers as mere advertising.

Therefore, given the existing evidence about the relationship between ICT, and the positive effect that it can generate on consumer perception of actions related to sustainability developed by companies, we propose the second hypothesis of this research:

H2: *The use of ICT in retail has a positive and significant effect on customer perceptions of ICT.*

#### Relationship Between Innovation and Sustainability

Some studies point out the importance of taking action in the main dimensions of innovation (product, process, organizational, marketing, and relationship) as the primary elements to achieve the development of sustainable business models (Gonzalez-Lafaysse and Lapassouse-Madrid, 2016; Marcon et al., 2017). Innovative business models for sustainability are defined as innovations that can generate significant negative or positive impact and/or reductions for the environment and/or society. In this way, the relationship between innovation and sustainability will allow, among other aspects, an increase in the competitiveness of companies through cost reduction, improved reliability and a better response to market needs. In addition, the introduction of innovative sustainable practices in retail formats is associated with high levels of consumer satisfaction, which facilitate greater customer retention.

Marcon et al. (2017) empirically contrast how some multinational companies have managed to adopt more sustainable practices in their businesses by implementing innovations in the products sold and in the processes carried out to develop those products. In addition, these sustainable practices have been integrated into the organizational structure of companies and in the marketing actions taken by these organizations. Therefore, Marcon et al. (2017) describe how product, process, organizational and marketing innovations contribute to facilitating changes toward more sustainable business models, taking the environmental dimension as a perspective.

Taking into account each of the dimensions of innovation and examining its impact on the development of sustainable actions, product innovation can have positive effects on the environment, by making use of less harmful materials, as a consequence of the knowledge and technology applied to the products developed (Gonzalez-Lafaysse and Lapassouse-Madrid, 2016). Second, process innovation allows companies to reduce costs (economic, social and environmental), raise the standard of quality and increase the supply of products and services (Kumar et al., 2017). Regarding organizational innovations, these are responsible for modifying or changing the relationships and decisions of companies. Training in sustainable innovation, cooperation with agents and stakeholders, business model innovation and consumer awareness are just some of the sustainable practices derived from relational innovations (Marcon et al., 2017). Finally, marketing innovations allow companies to develop new and more conscientious business practices (change in product design, store distribution, communication) in regard to the environment and society (Marín-García et al., 2020).

In this way, given the existing evidence about the relationship between innovation and sustainability, and the positive effect that it can generate on the perception of consumers on companies, we propose the third hypothesis of this research:

H3: *Customer perception of innovative retailer practices has a positive and significant effect on customer perception of sustainable in-store practices.*

#### The Moderating Effect of Store Format

In the context of retailing, there are few studies that address the moderating effect of store format on consumer perceptions of the degree of technological advancement of companies, innovation and sustainability. However, some research shows this effect on other variables traditionally linked to retail formats and which are currently strongly linked to ICT, innovation, and sustainability.

In this sense, Filipe et al. (2017) point out that the image of the store may be moderated by retail format, differentiating between small supermarkets and large supermarkets. The authors of the study start from the premise of the existence of two types of behavior according to the profile of the consumer (Piacentini et al., 2001): on the one hand, those consumers who decide to make their purchases in retail formats is near their home or workplace, and on the other, a group of customers who travel long distances to carry out the purchase process, attracted by external factors such as price discounts. Likewise, Filipe et al. (2017) point out that many consumers no longer patronize large stores as they are attracted to the personal treatment they receive in traditional stores. In the empirical analysis carried out by these authors, the moderating effect of store format is concluded, with significant differences between small and large supermarkets. In this sense, the authors point out that the small innovations carried out by traditional stores, and which are aimed at making the purchase process a warm and intimate experience for the consumer, also serve to improve the image of small stores.

Catuogno and Gutierres (2015), along the same lines as the previous authors, state that traditional store formats can be perceived as friendlier by consumers. In this sense, consumers of small store formats present higher levels of satisfaction toward this type of retail format than the segment of large-format customers. However, the evidence found by Graciola et al. (2020) is contrary to previous findings. These authors conclude that image acts as an independent variable and is not influence by any specific store format.

For all the above, observing the role that store format plays in the chain of effects contemplated can be an opportunity for research. Thus, given the existing empirical evidence in favor of the moderating effect of store format on consumer perceptions, we formulate the following group of hypotheses that make up the fourth hypothesis of this research:

H4a: *The influence of the use of ICT on customer perceptions of innovative practices in the establishment is moderated by store format.*

H4b: *The influence of the use of ICT on customer perceptions of sustainable practices in the establishment is moderated by store format.*

H4c: *The influence of customer perceptions of innovative practices in the store on customer perceptions of sustainable practices in the establishment is moderated by store format.*

#### The Mediating Effect of Innovation

Despite the importance that the study of innovation is acquiring, in the retail context, there are still many aspects to be analyzed, especially from the consumer perspective. From the point of view of companies, some studies point out the capital importance of innovation as a mediator between strategic orientation and business performance, in the sense that in those companies that implement innovative actions, strategic orientation leads to better financial results (Medina and Rufín, 2009). In this way, actions derived from innovation are considered a fundamental strategic factor in the progress of companies.

On the other hand, Pantano and Gandini (2017) examine the mediating effect of innovation in retailing by adopting the consumer perspective. The authors start from the premise that the retail environment is characterized by a growing use of advanced and interactive technologies, where mobile applications, virtual reality and augmented reality, among others, play a fundamental role. In this sense, the authors state that businesses that apply ICT, and use different tools from those used by competitors, are positively perceived by consumers, especially by the young segment. In this sense, the authors point out the important role of innovation in enriching the customer’s shopping experience. In other words, in order to provide new experiences to consumers, it is important not only to implement ICT, but also ensure that they are innovative.

Based on this reasoning, we postulate the final hypothesis of this study:

H5: *The effect of ICT on customer perceptions of sustainable practices in the store is mediated by customer perception of innovative practices in the store.*

**FIGURE 1:**
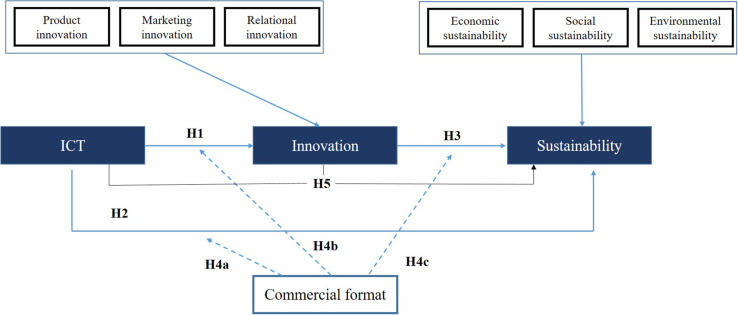
Proposed research model.

After formulating the hypotheses of this study, Figure 1 presents the structural model of this research.

## Methodology

In order to obtain the necessary information to test the previously stated hypotheses, a structured face-to-face survey was carried out with 510 individuals in the province of Valencia (Spain), following a non-probability quota sampling method. The retail formats have been retained based on the products offered and their positioning in the Spanish market, within the food sector, and serving the hypermarkets, supermarkets, and discount stores formats. Table 1 reflects the distribution of the sample.

**TABLE 1:** Sample distribution: sociodemographic variables.

	**Total**		**Hypermarket**		**Supermarket**		**Discount store**	
	**N**	**%**	**N**	**%**	**N**	**%**	**N**	**%**
**Gender**								
Male	206	40.4	68	40.0	67	39.4	71	41.8
Female	304	59.6	102	60.0	103	60.6	99	58.2
**Age**								
18–25 years old	36	7.1	16	9.4	9	5.3	11	6.5
26–35 years old	88	17.3	34	20.0	20	11.8	34	20.0
36–45 years old	133	26.1	35	20.6	63	37.1	35	20.6
46–55 years old	113	22.2	18	10.6	50	29.4	45	26.5
56–65 years old	108	21.2	50	29.4	24	14.1	34	20.0
Over 65 years old	32	6.3	17	10.0	4	2.4	11	6.5
**Education**								
No schooling	40	7.8	20	11.8	9	5.3	11	6.5
Primary education	84	16.5	41	24.1	22	12.9	21	12.4
Secondary education	148	29.0	41	24.1	46	27.1	61	35.9
University studies	238	46.7	68	40.0	93	54.7	77	45.3
**Occupation**								
Employee	282	55.3	90	52.9	102	60.0	90	52.9
Employer/Self-employed	63	12.4	16	9.4	24	14.1	23	13.5
Pensioner	58	11.4	39	22.9	6	3.5	13	7.6
Unemployed	40	7.8	6	3.5	16	9.4	18	10.6
Housewife	37	7.3	16	9.4	12	7.1	9	5.3
Student	30	5.8	3	1.8	10	5.9	17	10.0

Total	510		170		170		170	

For data collection, an *ad hoc* structured questionnaire with closed-ended questions was distributed, in which respondents had to indicate their degree of agreement or disagreement with a series of statements. Specifically, the questionnaire collected information on the customer perception of innovation in stores, assessing the level of technological advancement in stores and their perception of innovative and sustainable practices implemented in retail stores. Additionally, in order to segment the sample obtained, some classification questions were included, based on sociodemographic criteria. The items related to the different variables analyzed have been extracted and adapted from various scales used in the marketing literature, which have proven content validity. Specifically, the items to measure innovation in stores have been adapted from the Lin (2015) scales; for ICT, items were adapted from the proposal by Wu et al. (2006), in order to evaluate to what extent consumers perceive the technological advances of the company. Finally, sustainability was measured using the scale proposed by Lavorata (2014). In all cases, the items were measured using a 7-point Likert scale (see Appendix).

For the analysis of the data obtained we decided to proceed in two stages. Firstly, the measurement instrument was validated; and, secondly, the structural model was estimated. Both analyses were performed using the partial least squares (PLS) regression technique. It is a technique that in recent years has been widely used by academics and researchers (Evermann and Tate, 2016; Henseler et al., 2016; Henseler, 2017). One of the main characteristics that must be assessed in the systematic process for the application of the PLS-SEM analysis is that it is a non-parametric statistical procedure, in which it is not necessary for the data to fit a normal distribution (Hair et al., 2016).

## Results

### Reliability and Validity of the Measuring Instrument

Before examining the reliability and validity of the scales used, the dimensionality of the second-order constructs was analyzed. For this, an Exploratory Factor Analysis was performed using the Principal Component Analysis technique with Varimax rotation. The result of this analysis confirmed the multidimensionality of innovation and sustainability. In this way, on the one hand, the results obtained indicated that innovation is a construct formed from three factors (product innovation, marketing innovation and relational innovation), confirming what is postulated by Lin (2015). The three factors together explain 68.93% of the total variance. Similarly, sustainability was confirmed as a construct formed from three dimensions: economic sustainability, social sustainability, and environmental sustainability, in the line of Lavorata (2014). Together, the three sustainability factors explain 72.91% of the total variance.

Next, we proceeded to analyse the reliability and validity of the measurement instrument; we observed for each of the constructs of this study the factor loadings, Cronbach’s alpha, the composite reliability index and the average variance extracted (AVE). For this, the PLS-SEM technique was used and a PLS algorithm of 300 maximum interactions and a path weighting scheme were calculated. The results of these analyses are presented in Table 2. On the other hand, items with values lower than 0.7 are eliminated (Bagozzi and Yi, 1988), deleting an indicator from the ICT scale.

**TABLE 2:** Measurement instrument of the structural model: Reliability and convergent validity.

**Factor**	**Item**	**Loading**	**t**	**Cronbach α**	**CR**	**AVE**
ICT	ICT1	0.895***	80.658	0.915	0.946	0.855
	ICT2	0.938***	140.579			
	ICT3	0.940***	159.603			
Economic sustainability	SB1	0.800***	31.039	0.817	0.890	0.729
	SB2	0.901***	84.714			
	SB3	0.858***	49.563			
	SB4	0.835***	38.478			
Social sustainability	SB5	0.741***	28.401	0.883	0.913	0.679
	SB6	0.888***	88.008			
	SB7	0.800***	32.192			
	SB8	0.814***	34.996			
	SB9	0.869***	61.931			
Environmental sustainability	SB10	0.824***	31.831	0.817	0.890	0.729
	SB11	0.881***	57.452			
	SB12	0.856***	42.326			
Product innovation	IN1	0.848***	41.802	0.860	0.899	0.641
	IN2	0.720***	19.558			
	IN3	0.833***	39.627			
	IN4	0.787***	33.182			
	IN5	0.808***	35.197			
Marketing innovation	IN6	0.723***	25.374	0.890	0.916	0.647
	IN7	0.765***	33.886			
	IN8	0.853***	63.787			
	IN9	0.829***	19.558			
	IN10	0.850***	70.108			
	IN11	0.798***	34.847			
Relational innovation	IN12	0.838***	39.990	0.866	0.918	0.790
	IN13	0.910***	79.850			
	IN14	0.917***	102.120			

Statistically significant at ***p < 0.01.

The results obtained allow us to confirm the reliability and convergent validity of the measurement instrument. On the one hand, the Cronbach’s α values presented in Table 2 far exceed the reference value 0.7 (Churchill, 1979; Nunnally and Bernstein, 1994). Specifically, the results obtained show values that range between 0.8 and 0.9, identified as recommended values for advanced stages of research (Nunnally and Bernstein, 1994). On the other hand, the composite reliability presents satisfactory values that exceed 0.7, the minimum required index (Chin, 1998). Furthermore, in relation to the average variance extracted, the results obtained are greater than 0.5, which means that each construct explains at least 50% of the variance of the established indicators (Fornell and Larcker, 1981). Finally, to confirm the discriminant validity of the variables that form the structural model of this study, we used the criterion of Fornell and Larcker (1981). The results of this analysis show that the square root of the AVE is higher than the estimated correlation between the factors for all cases (Table 3).

**TABLE 3:** Measurement instrument: Discriminant validity (Fornell-Larcker criterion).

	**1**	**2**	**3**	**4**	**5**	**6**	**7**
1. Marketing innovation	**0.804**						
2. Relational innovation	0.581	**0.889**					
3. Product innovation	0.602	0.423	**0.800**				
4. Economic sustainability	0.463	0.210	0.319	**0.849**			
5. Environmental sustainability	0.341	0.275	0.258	0.514	**0.854**		
6. Social sustainability	0.440	0.307	0.365	0.575	0.611	**0.824**	
7. ICT	0.639	0.393	0.341	0.530	0.360	0.420	**0.925**

*Diagonal (bold values): square root of average variance extracted (AVE). Below the diagonal: correlations between factors.*

Additionally, when considering innovation and sustainability as second-order formative constructs, the weightings of the respective factors were examined. Following the criteria established by Diamantopoulos and Winklhofer (2001), the absence of collinearity was verified through the variance inflation factor (VIF). The result obtained from this analysis corroborates the absence of collinearity, since the VIF values for each of the dimensions of innovation and sustainability are below the critical level of 5 (Table 4).

**TABLE 4:** Parameter estimates of the formative second-order constructs.

**1st Level Factor**	**2nd Level Factor**	**Weight**	**VIF**
Sustainability	Economic sustainability	0.448***	1.594
	Social sustainability	0.404***	1.871
	Environmental sustainability	0.333***	1.701
Innovation	Product innovation	0.330***	1.590
	Marketing innovation	0.530***	1.970
	Relational innovation	0.327***	1.529

Statistically significant at ***p < 0.01.

### Evaluation of the Structural Model

Later, after confirming the reliability and validity of the measurement instrument, the structural model was tested. To do this, following the criteria of Ringle et al. (2015), the bootstrap method was used with 5000 subsamples. This procedure allowed us to evaluate the relationships proposed in this research, analyse the significance of the hypotheses raised and obtain the results of the *R*^2^ values of the explained variance. Furthermore, using the blindfolding technique, the predictive relevance of the *Q*^2^ test was examined. The results obtained from the estimation of the structural model are presented in Table 5.

**TABLE 5:** Causal relationships estimation.

**Relationship**		**Total sample**			**Hypermarket**	**Supermarket**	**Discount stores**
		**Hypothesis**	**Standardized parameter**	**t**	**Standardized parameter**	**Standardized parameter**	**Standardized parameter**
H1	ICT → Innovation	Supported	0.580***	24.462	0.547***	0.540***	0.322***
H2	ICT → Sustainability	Supported	0.366***	7.337	0.185ns	0.425***	0.348***
H3	Innovation → Sustainability	Supported	0.277***	5.857	0.332*	0.278***	0.223**

*Innovación: R^2^* = *0.336, Q^2^* = *0.210; Sostenibilidad: R^2^* = *0.328, Q^2^* = *0.217.* * Statistically significant at *p < 0.1; **p < 0.05; ***p < 0.01; ns = statistically non-significant.

The results of the PLS-SEM analysis allow us to confirm the first three relationships of the structural model of this work. In this sense, the result of the first two hypotheses confirms ICT as a driving force for innovation and sustainability in retailing by validating the positive and significant relationship between ICT and both constructs (β1 = 0.580, *p* < 0.001; β2 = 0.366, *p* < 0.001), the effect of ICT being higher in innovation than in sustainability. In addition, from the results obtained it is possible to confirm the positive and significant nature of the innovation-sustainability relationship (β3 = 0.277, *p* < 0.001).

### Analysis of the Moderating Effect of the Retail Format

On the other hand, in order to test the group of hypotheses that make up the fourth hypothesis of this research, a multigroup analysis was carried out using the PLS-MGA technique. The results presented in Table 6 show the moderating effect of store format on the relationship between ICT and innovation for hypermarkets versus discount stores, and for supermarkets versus discount stores. In contrast, there are no significant differences between hypermarket and supermarket consumers in the relationships proposed. However, it is possible to affirm that the relationship between ICT and innovation is moderated by store format, in view of the differences between discount stores and the rest of the establishments under study. These results are aligned with those studies that suggest that, when making decisions in retailing, it is very relevant to consider under which store format to compete.

**TABLE 6:** Results of the multigroup analysis.

**Hypothesis**	**Relationship**	**Hypermarket-Supermarket Stand.parameter**	***p*-value**	**Hypermarket –Discount store Stand.parameter**	***p*-value**	**Supermarket- Discount store Stand.parameter**	***p*-value**
H4a	ICT → Innovation	0.007ns	0.453	0.225**	0.003	0.217**	0.002
H4b	ICT → Sustainability	0.240ns	0.890	0.163ns	0.807	0.077ns	0.255
H4c	Innovation → Sustainability	0.054ns	0.300	0.110ns	0.206	0.055ns	0.299

Statistically significant at **p < 0.05; ns = statistically non-significant.

### Analysis of the Mediating Effect of the Innovation

Finally, the analysis of the role of innovation as a mediating variable between ICT and sustainability in retailing was carried out through Preacher and Hayes′ (2008) bootstrapping technique. Table 7 shows that there is a significant direct and indirect effect between ICT and sustainability. For this reason, there is support for hypothesis H5, which postulates the mediating role of innovation in the ICT-Sustainability relationship in retailing. Additionally, we can confirm through the result of the Variance Accounted For (VAF), which determines the size of the indirect effect in relation to the total effect (Hair et al., 2014; Nitzl et al., 2016; Cepeda et al., 2017), that innovation has a partial mediating effect in this relationship (0.310).

**TABLE 7:** Summary of mediating effect test.

**Relation**	**Total effect**	**Direct effect**	**Indirect effect**	**VAF**
ICT → Sustainability	0.527***	0.367***	0.160***	0.310

Statistically significant at ***p < 0.01.

Furthermore, in Table 7 we can observe the direct and indirect effect of the mediation of innovation. In this sense, it is observed that the relationship between ICT and sustainability would be positive for those commercial formats that make use of innovative actions in their businesses, while the relationship between sustainability and ICT will not be so positive for organizations with a low implementation of innovative practices.

## Discussion

This work has tried to continue the line of study of those investigations that indicate the importance of progressing in retailing through the development of innovative and sustainable actions (Gonzalez-Lafaysse and Lapassouse-Madrid, 2016; Morioka et al., 2016; Marcon et al., 2017; Marín-García et al., 2020, 2021). In this sense, this study advances in the knowledge of these constructs, incorporating ICT in the same causal model, and examining the differences in consumer perceptions about these practices, depending on the retail format of which they are customers. Thus, this work has set out to examine the impact of ICT on sustainability, contemplating both direct and mediated effects through innovation, and to show whether these effects in the chain of consequences are modified according to store format. Through this study, consumer perceptions have been examined in relation to the degree of technological advancement of companies, and the implementation of innovative and sustainable actions in retail formats. A better understanding of consumer buying behavior will facilitate the decision-making of retail business managers. In the following sections we will present the main theoretical conclusions, implications for management and the main limitations, which can be considered as future lines of research.

## Theoretical Conclusion

The results obtained reveal relevant findings that allow to progress in the knowledge related to the postulated chain of consequents. All of them are presented below.

Firstly, the evidence obtained allows us to conclude that, in retailing, ICT can be considered as a driving force for innovation and sustainability. In this way, the degree of consumer perception of the technological advancement of retail companies has a direct impact on the perceptions that customers have about innovative practices and sustainable actions implemented by retail formats. These results reflect the importance of ICT in retailing, as they have the power to trigger positive effects both in innovation (Pantano and Gandini, 2017) and in sustainability (De Vass et al., 2020).

Secondly, through this research support is found for the multidimensionality of innovation and sustainability as perceived by consumers, both constructs formed from three dimensions each. Thus, innovation in retailing under a market approach can be conceptualized as a second-order construct formed by product innovation, marketing innovation, and relational innovation, in the line of Lin (2015). Furthermore, sustainability can be considered as a construct formed by the three factors identified by the TBL model (Elkington, 2004): economic sustainability, social sustainability, and environmental sustainability.

Thirdly, our results show that, given the character of partial mediator of innovation (Pantano and Gandini, 2017), this construct is a determining variable in business strategy, since it provides substantive information on how ICT act on consumer perception of sustainability actions implemented in the retail formats. Perceptions regarding the degree of technological advancement of the retailer generate direct and mediated effects, through in-store innovation, on sustainability in retail. Consequently, this work contributes to the existing literature by providing information about how and why the effect of ICT perceptions on sustainable practices occurs, insofar as it is the inclusion of innovation in the equation, which intensifies its explanatory power. In addition, unlike what authors such as Gonzalez-Lafaysse and Lapassouse-Madrid (2016) point out, this work does observe positive perceptions of innovative and sustainable actions by consumers toward retail formats, regardless of the retail format of which they are customers.

Finally, the moderating role of store format revealed in this work is a conclusion of special interest, since it highlights the need to consider the characteristics of the retail formats, in terms of commercial concept, in the analysis of consumer behavior in the retail sector, following what was previously postulated by Filipe et al. (2017). Thus, the results show that the intensity of the observed relationships is affected by retail format, highlighting notable differences between the discount stores and the other store formats studied in this research, namely hypermarkets and supermarkets, especially when considering how ICT boosts innovation, observing that its effect is much higher in hypermarkets and supermarkets. Therefore, through this research we can conclude that consumer perception in regard to the use of ICT and innovative practices in retail formats is strongly linked to store format.

## Managerial Conclusion

In addition to the contributions described, the results obtained in this research permit the development of a set of implications for the management of large-scale food distribution companies. Firstly, the importance of ICT, due to their influence on innovation and sustainability, points to the need for companies to incorporate tools related to technological progress. This fact is relevant since it is possible to affirm that innovation, sustainability and ICT are walking in the same direction. In this sense, ICTs constitute the material evidence of innovation and sustainability, that is, they are the tools that make innovation and sustainability tangible in the eyes of the client.

In this sense, companies can rely on AI to increase consumer satisfaction and improve their perception of innovative and sustainable practices. The introduction of “independent recommendation showcases,” through which customers can receive a more customized offer, enhances the consumer’s shopping experience. In this sense, Artificial Intelligence allows the recommendations received by consumers to be as personalized as possible, without the need for periodic configurations, simply through the analysis of the consumer profile. In addition, businesses could make use of the so-called “smart search,” similar to the systems already used by Google or Bing. In this way, AI makes it possible to speed up the search for products by recognizing the similarity between the sounds of the letters and showing the results even with spelling or grammatical errors, or with image recognition. On the other hand, the use of “chatbots” is yet another tool that is transforming businesses. Through this support, customers can obtain accurate and immediate information 24 h a day, seven days a week through chats accessible within the microsites. Tools such as those presented can help consumers perceive retail formats as more innovative and sustainable. Companies should pay greater attention to the tools derived from ICT, especially those that have a digital nature, to facilitate these innovative processes, in the line of Gil-Saura et al. (2014) and Marcon et al. (2017). Furthermore, it seems clear that innovative companies are more prone to the use and application of ICT in their businesses (Musso, 2010; Gil-Saura et al., 2014). As a consequence of the positive effects between innovation and ICT that derive from this research, the need to increase support from retail managers on marketing policies also seems obvious. This support can be expressed through increased investment in this area, especially in digital media.

Secondly, the evidence obtained demonstrates the importance of incorporating innovative actions in retail formats to achieve high levels of perception related to the sustainability of businesses. In this sense, the development of products using materials that are less harmful to the environment, or communication and promotion campaigns through collaboration with non-profit associations, can be key decisions for consumers to perceive that retail formats operate from a dual perspective: innovative and sustainable. Following what was previously postulated by Kumar and Polonsky (2019), some of the measures that could be incorporated to make retail businesses more innovative and sustainable spaces are: the distinction of ecological products, the promotion of sustainable commercial practices and the availability and visibility of organic products. From our point of view, we believe that large food retailers could improve sustainability results, in the eyes of the consumer, through the commercialization of fair trade products and by improving the salaries of their employees and the prices they pay to their suppliers. However, for this type of action to be successful it is necessary that the decisions are not taken unilaterally by companies but that they actively seek consumer input. In this sense, the companies’ willingness to listen to consumer opinion is an essential element for the development of innovative and sustainable actions in retail formats.

Based on the above, we propose that organizations rely on ICTs and innovation as instruments that facilitate and promote initiatives of a sustainable nature. The development of products, using materials that are less harmful to the environment, or increasing the presence on the shelves of products such as those that come from fair trade, are actions that will improve the image that consumers have toward companies, in addition to contribute to perceived quality and loyalty to them. Likewise, use new knowledge and processes in the production and manufacture of products and services, new forms of organization that integrate the concept of sustainability, corporate responsibility and ethics into their strategic plans, or changes in retail formats, store concept or in Relationships with consumers also contribute to improving the image, quality, notoriety and loyalty of retail companies (Marín-García et al., 2020). Therefore, we propose that companies increase their investments in innovation and ICT, among others those aimed at saving energy to facilitate the implementation of sustainability policies in their establishments.

## Limitations and Future Lines of Research

However, it is important to note that there are a number of limitations, which open up new future research opportunities. Firstly, in relation to the variables that make up the research model, it is important to make progress in the development of scales linked to ICT that have as a base pillar the analysis of the tools associated with AI. In this sense, we consider that a better adjustment of the ICT scale based on AI is key to a better understanding of consumer perceptions of innovative and sustainable activities implemented by retailers. Second, this research does not cover all the proposals for existing store formats; therefore, it would be interesting to further develop its analysis by including new types of retail formats. Furthermore, the study of other moderating variables, of a sociodemographic nature, such as customer age, level of education or main occupation, may help to explain how ICT and innovation intensify positive perceptions toward sustainable practices. Additionally, the study of the differences in the perceptions that consumers from other regions have toward sustainable practices of retailers and whether these differences may be due to aspects such as culture, lifestyle or commercial structure, could also constitute a future line of research. Finally, this analysis could also be extended to examine the differences between the brands used in this research, or the study of retail sectors other than food.

## Data Availability Statement

The raw data supporting the conclusion of this article will be made available by the authors, without undue reservation.

## Ethics Statement

Ethical review and approval was not required for the study on human participants in accordance with the local legislation and institutional requirements. The patients/participants provided their written informed consent to participate in this study.

## Author Contributions

All authors listed have made a substantial, direct, and intellectual contribution to the work and approved it for publication.

## Conflict of Interest

The authors declare that the research was conducted in the absence of any commercial or financial relationships that could be construed as a potential conflict of interest.
