# Supplementary material for: The Moderating Effect of Store Format on the Relationships Between ICT, Innovation and Sustainability in Retailing
Source: Front Psychol. 2021 May 6;12:678991. doi: 10.3389/fpsyg.2021.678991 (PMC8134666; doi:10.3389/fpsyg.2021.678991)
Supplement: Supplementary file 1 [file Data_Sheet_1.docx]

**Appendix**

| Factor | Item | Description | Hypermarket | | Supermarket | | Discount store | | Total | |
| --- | --- | --- | --- | --- | --- | --- | --- | --- | --- | --- |
|  |  |  | Mean | σ | Mean | σ | Mean | σ | Mean | σ |
| ICT | ICT1 | STORE X invests in technology | 5.30 | 1.685 | 4.81 | 1.141 | 3.46 | 1.350 | 4.52 | 1.609 |
|  | ICT2 | The ICT of STORE X are always the latest in technology | 5.10 | 1.190 | 4.39 | 1.203 | 3.48 | 1.315 | 4.32 | 1.403 |
|  | ICT3 | Compared to its competitors, STORE X technology is more advanced | 5.59 | 1.420 | 4.38 | 1.324 | 3.28 | 1.256 | 4.42 | 1.634 |
|  | ICT4 | This STORE X takes into account my opinion as a client to coordinate and develop ICT in order to improve the service and better satisfy my needs as a client | 3.64 | 1.701 | 4.21 | 1.464 | 3.55 | 1.328 | 3.80 | 1.531 |
| Economic sustainability | SB1 | STORE X pays producers a fair price | 4.42 | 0.921 | 4.07 | 1.290 | 3.82 | 0.953 | 4.10 | 1.094 |
|  | SB2 | STORE X pays its employees a decent wage | 4.52 | 1.028 | 4.68 | 1.001 | 3.15 | 1.007 | 4.11 | 1.224 |
|  | SB3 | STORE X pays its employees a minimum wage in developing countries | 4.37 | 0.996 | 4.20 | 1.175 | 3.61 | 0.993 | 4.06 | 1.106 |
|  | SB4 | STORE X monitors the working conditions of its employees | 4.18 | 0.795 | 4.66 | 1.187 | 3.51 | 1.132 | 4.11 | 1.152 |
| Social sustainability | SB5 | STORE X sells fair trade products | 4.64 | 1.652 | 3.82 | 1.582 | 3.57 | 0.960 | 4.01 | 1.500 |
|  | SB6 | STORE X sells organic products | 5.45 | 1.827 | 4.46 | 1.550 | 4.29 | 1.139 | 4.73 | 1.611 |
|  | SB7 | STORE X implements humanitarian actions | 4.72 | 1.407 | 4.60 | 1.655 | 4.44 | 1.093 | 4.59 | 1.406 |
|  | SB8 | STORE X engages in actions directed at schools | 4.58 | 1.170 | 4.82 | 1.651 | 4.31 | 1.050 | 4.57 | 1.330 |
|  | SB9 | STORE X sells share products (donations to charitable associations) | 4.93 | 1.619 | 4.99 | 1.521 | 4.28 | 1.296 | 4.73 | 1.517 |
| Environmental sustainability | SB10 | STORE X recycles their products and packaging | 4.25 | 0.936 | 4.45 | 1.460 | 4.20 | 1.175 | 4.30 | 1.212 |
|  | SB11 | STORE X cuts back their consumer of electricity | 4.38 | 0.737 | 4.45 | 1.410 | 4.12 | 1.010 | 4.32 | 1.095 |
|  | SB12 | STORE X pays attention to the environment | 4.88 | 0.998 | 4.79 | 1.423 | 4.27 | 0.909 | 4.65 | 1.162 |
| Product innovation | IN1 | Offers many new products | 5.61 | 1.629 | 4.42 | 1.605 | 4.45 | 1.115 | 4.83 | 1.566 |
|  | IN2 | Offers creative own designed products | 5.05 | 1.944 | 3.81 | 1.576 | 3.95 | 1.056 | 4.27 | 1.661 |
|  | IN3 | Offers innovative private brand products | 5.34 | 1.668 | 4.58 | 1.522 | 3.91 | 1.067 | 4.61 | 1.554 |
|  | IN4 | Offers more innovative products than other stores | 5.57 | 1.671 | 3.87 | 1.498 | 3.93 | 1.159 | 4.46 | 1.655 |
|  | IN5 | Offers various products for selection | 5.77 | 1.675 | 4.42 | 1.612 | 4.39 | 1.173 | 4.86 | 1.633 |
| Marketing innovation | IN6 | Offers many innovative self-services | 5.20 | 1.525 | 4.42 | 1.426 | 4.12 | 1.203 | 4.58 | 1.461 |
|  | IN7 | Offers many innovative services | 5.96 | 1.329 | 4.36 | 1.317 | 3.91 | 1.338 | 4.74 | 1.592 |
|  | IN8 | Offers more innovative services than other stores | 5.89 | 1.399 | 4.89 | 1.197 | 4.07 | 1.041 | 4.95 | 1.428 |
|  | IN9 | Creates a holiday atmosphere through store decoration | 6.08 | 1.153 | 4.48 | 1.403 | 3.80 | 0.868 | 4.79 | 1.504 |
|  | IN10 | Has ability to create a different in-store atmosphere | 5.24 | 1.271 | 4.35 | 1.360 | 3.46 | 1.121 | 4.35 | 1.448 |
|  | IN11 | Offers an innovative shopping environment | 5.06 | 1.636 | 4.01 | 1.427 | 3.48 | 1.110 | 4.18 | 1.552 |
| Relational innovation | IN12 | Offers a creative store design | 6.27 | 1.225 | 4.83 | 1.814 | 5.01 | 1.156 | 5.37 | 1.564 |
|  | IN13 | Offers different discount programs | 5.25 | 1.538 | 4.38 | 1.737 | 4.75 | 1.277 | 4.79 | 1.567 |
|  | IN14 | Offers an innovative promotion mix | 5.33 | 1.763 | 4.29 | 1.901 | 4.46 | 1.283 | 4.69 | 1.728 |
